# The Cost of Vice

**Published:** 1914-07

**Authors:** 


### The Cost of Vice.

Speaking of the State's regulation of social diseases from an economic standpoint, it is interesting to know something of the cost of this vice to the country. While statistics are often unreliable and at best only indicate averages, the figures below underestimate rather than overestimate the true situation.

Howard Kelley estimates cost of venereal diseases to America at three billion dollars annually; $125,000,000 are spent alone in illicit sexual congress. The Chicago Vice Commission places the cost to that city at $15,000,000 annually. Flexner estimates the cost of prostitution to the German Empire as from $75,000,000 to $125,000,000 annually, and contrasts this huge expenditure to the amount the government spends on its educational system. In 1909 the educational budget, including universities, secondary schools, elementary schools and technical and professional institutions, was approximately $50,000,000 a year. He very pointedly remarked, "assuredly the economic burden imposed on society by prostitution is comparable with that due to standing armies, war or pestilence."

